# A Nomogram to Predict Disease-Free Survival Following Neoadjuvant Chemotherapy for Triple Negative Breast Cancer

**DOI:** 10.3389/fonc.2021.690336

**Published:** 2021-10-21

**Authors:** Meizhen Zhu, Chenlu Liang, Fanrong Zhang, Liang Zhu, Daobao Chen

**Affiliations:** ^1^ Department of Breast Surgery, The Cancer Hospital of the University of Chinese Academy of Sciences (Zhejiang Cancer Hospital), Institute of Basic Medicine and Cancer (IBMC), Chinese Academy of Sciences, Hangzhou, China; ^2^ Department of Pathology, The Cancer Hospital of the University of Chinese Academy of Sciences (Zhejiang Cancer Hospital), Institute of Basic Medicine and Cancer (IBMC), Chinese Academy of Sciences, Hangzhou, China

**Keywords:** nomogram, disease-free survival, neoadjuvant chemotherapy, triple negative breast cancer, prediction

## Abstract

**Background:**

Neoadjuvant chemotherapy (NACT) is considered a standard treatment strategy for locally advanced triple negative breast cancer (TNBC). TNBC patients who achieve a pathologic complete response (pCR) are predicted to have a better prognosis while unfavorable chemo-sensitivity is still associated with a higher risk of disease relapse. The objective of this study was to construct a nomogram to predict disease-free survival (DFS) for TNBC patients following NACT.

**Methods:**

A total of 165 TNBC patients who underwent standard NACT and surgery were retrospectively reviewed, and data on their clinicopathological factors before and after NACT were collected. Independent prognostic factors for DFS were identified by Cox regression based on lower Akaike information criteria (AIC) and Bayesian information criterion (BIC). A nomogram to predict the 2-year and 5-year DFS following NACT for TNBC was constructed based on training cohort (n = 132) and validated by a validation cohort (n = 33).

**Results:**

Either limited or full pCR (breast-only pCR, node-only pCR, or both-pCR) indicated significantly improved DFS and overall survival (OS) (p < 0.001). Lager residual tumor size (hazard ratio [HR] 1.175, p = 0.011) and the presence of lymphatic vessel invasion (LVI) (HR 3.168, p = 0.001) were identified as independent predictors of disease relapse in the training cohort. Five variables, including age, primary tumor size, histological grade, residual tumor size, and LVI were used to establish the nomogram. The C-index of the nomogram was 0.815, and calibration curves showed an acceptable consistency between the actual and nomogram-predicted 2-year and 5-year DFS. The proposed nomogram demonstrated superior predictive performance compared with Residual Cancer Burden (RCB) classification and the 8th American Joint Committee on Cancer Post Neoadjuvant Therapy Classification (AJCC ypTNM) staging system (area under the curve [AUC] for 2-year DFS: 0.870 *vs.* 0.758 *vs.* 0.711, respectively; AUC for 5-year DFS: 0.794 *vs.* 0.731 *vs.* 0.702, respectively) in the validation cohort.

**Conclusions:**

The nomogram proposed in our study enabled to quantify the risk of disease relapse and demonstrated superior predictive performance than a survival predict instrument. It was an easy-to-use tool for clinicians to guide individualized surveillance of TNBC patients following standard NACT.

## Introduction

For patients with triple negative breast cancer (TNBC), characterized by lack of expression of estrogen receptor (ER), progesterone receptor (PgR), and human epidermal growth factor receptor-2 (HER-2) amplification, chemotherapy remains the chief systemic treatment option because specific endocrine and molecular targets are unavailable (1). Neoadjuvant chemotherapy (NACT) has been accepted as a standard therapeutic strategy for locally advanced TNBC. Despite the aggressive clinical behavior of TNBC, approximately 30-40% of patients can achieve pathologic complete response (pCR) after NACT (2). pCR is a strong surrogate of better outcome for TNBC (3), and for patients with residual invasive disease after standard NACT based on a combination of anthracycline and taxane, adjuvant capecitabine therapy would be recommended to improve prognosis (4). Nevertheless, TNBC patients, especially those with an unfavorable response to NACT, are still associated with higher risk of early relapse, higher incidence of visceral metastases, and poorer outcome.

Recent studies have described the predictors of pCR (5), while few predictors that risk-stratify patients following NACT have been reported. Compared with pCR, the Residual Cancer Burden (RCB) classification system and American Joint Committee on Cancer Post Neoadjuvant Therapy Classification (AJCC ypTNM) system additionally distinguish patients with residual tumor (6, 7). The RCB system calculates a score based on the primary tumor bed area containing residual tumor, overall cancer cellularity, percentage of cancer that is considered *in situ* disease, number of positive lymph nodes, and the diameter of the largest metastasis (6). The AJCC ypTNM system considers three parameters for pathological staging: residual tumor in the breast (ypT), residual nodal involvement (ypN), and distant metastases (7). Since tumor, node, and metastasis are included, both the RCB and AJCC ypTNM systems are preferred for pathologic evaluation of post-neoadjuvant specimens and are commonly used as instruments to predict survival after NACT (8). However, there are still limitations, such as age, histological grade, lymphatic vessel invasion (LVI) and post neoadjuvant therapy that have not been taken into account for prognostic prediction. This indicates that currently no ideal multivariable predictor exists to estimate the recurrence probability after NACT.

Therefore, it is necessary to establish a more comprehensive and accurate prediction model to estimate individual risk for clinical decision-making. Nomograms are graphical and easy-to-use models that enable users to calculate the probability of a clinical event for an individual patient (9). Based on the combination of clinical and pathological variables, the aim of the present study was to establish and validate a nomogram capable of predicting disease-free survival (DFS) of TNBC patients following NACT, which in turn can be used to guide individualized post-neoadjuvant surveillance.

## Materials and Methods

### Patients

Breast cancer patients that received NACT between January 2015 and December 2018 at our center were retrospectively reviewed. The inclusion criteria were as follows: (1) female sex and primary unilateral breast cancer; (2) pathologically confirmed TNBC before NACT; (3) receiving standard NACT regimen (doxorubicin/cyclophosphamide followed by docetaxel every 3 weeks); (4) subjected to surgical resection with axillary lymph node dissection. Patients were excluded with any of the following: (1) history of previous or concurrent cancer; (2) evidence of distant metastases; (3) receiving less than two cycles of NACT; (4) surgical specimens after NACT confirmed as non-TNBC resulting from molecular changes. TNBC was defined as negative for ER and PgR if less than 1% of cells showed positivity by immunohistochemical staining, and HER-2 negative determined by the American Society of Clinical Oncology (ASCO)/College of American Pathologists (CAP) guidelines (10).

This study was approved by the Clinical Research Ethics Committee of Zhejiang Cancer Hospital and all patients provided written informed consent (IRB-2020-327) before participation.

### Clinicopathological Variables

Clinical data included age, menopausal status, and family history. Primary tumor size was measured by breast magnetic resonance imaging (MRI) based on the longest tumor diameter. Axillary lymph node involvement was confirmed by fine needle aspiration. Then patients were staged according to the 8th edition of the AJCC TNM staging system (7). Tumor response was assessed according to version 1.1 of Response Evaluation Criteria in Solid Tumors (RECIST), evaluated as a complete response (CR), partial response (PR), stable disease (SD), or progressive disease (PD) (11). NACT cycles, surgery types and post neoadjuvant therapy were recorded. Standard intensive treatment of capecitabine after NACT was defined as at least 6 cycles of capecitabine (1250 mg/m^2^, twice a day, 14 days a cycle).

Pathological data included histological type, histological grade, Ki-67, and androgen receptor (AR) status before NACT. Ki-67 was defined as low expression when the fraction of positively stained cells was ≤29%, and high when the positively stained fraction was >29% according to our pathological laboratory center. AR was defined as positive when the percentage of tumor nuclei staining >1% (12). Surgical specimens after NACT were thoroughly examined using standard protocols. Each of breast specimen was divided into parallel sections at intervals of 5-10 mm, and the lesions on each section were carefully measured. The lymph nodes were sectioned in parallel with an interval not exceeding 2 mm, and each tissue was carefully examined. Residual tumor size, pathological T category (ypT), N category (ypN), pathological LVI post-neoadjuvant therapy were recorded. pCR was defined as residual ductal carcinoma *in situ* or complete disappearance of all invasive carcinoma cells in the breast and axillary lymph nodes (ypT0/is ypN0) (13). All specimens were evaluated by RCB and AJCC ypTNM criteria.

### Follow-up

All patients were recommended to receive regular follow-up visits after completion of treatment according to clinical guidelines (14). Patients were generally followed up every 3 months for the first 2 years and every 6 months thereafter if no evidence of relapse had occurred. At each follow-up, laboratory and imaging examinations were performed, including ultrasound for local (chest wall), regional (lymph node) and abdominal examinations, chest computed tomography for monitoring lung metastases, brain MRI for monitoring brain metastases, bone emission computed tomography (ECT) for monitoring bone metastases. DFS was defined as the time interval from diagnosis to recurrence (i.e., local, regional recurrence, or distant metastasis) or death for any reason. Overall survival (OS) was defined as the time interval from diagnosis to death for any reason. Surviving patients were followed up until 30 November 2020.

### Statistical Analysis

Statistical analysis was performed using SPSS software (version 25.0; IBM Corporation, Armonk, NY, USA) and R software (version 4.0.3; https://www.r-project.org/). DFS and OS curves were drawn using the Kaplan-Meier method and compared using the log-rank test. Then enrolled patients were randomly (4:1) divided into training and validation datasets. Categorical variables were analyzed using Chi-square test or Fisher’s exact test and continuous variables were analyzed using Student’s t test or the Mann-Whitney U test. Cox proportional hazards regression was used for univariable analysis related to disease relapse in the training dataset. Potential prognostic factors (p < 0.1) in the univariate analysis were included in the multivariate Cox regression analysis for selecting models based on lower Akaike information criteria (AIC) and Bayesian information criterion (BIC) to identify independent prognostic factors. All reported p values are two-sided, and p < 0.05 was considered statistically significant.

A nomogram for predicting the 2-year and 5-year DFS was constructed using the package of rms in R software (version 4.0.3; https://www.r-project.org/). The nomogram was quantified with respect to discrimination and calibration. Discrimination was quantified with the area under the receiver operating characteristic curve (AUC) or with the Harrell’s concordance index (C-index), which is considered to represent relatively good discrimination of the model if C-index is greater than 0.750. Calibration curves were performed by comparing the nomogram-predicted probability with the Kaplan-Meier estimator in the training and validation datasets. Receiver operating characteristic (ROC) curves for the 2-year and 5-year DFS were used to compare the proposed prediction model with the RCB and AJCC ypTNM system in the validation cohort.

## Results

A total of 165 consecutive TNBC patients who underwent NACT and surgery were retrospectively enrolled. Of these, 56 (33.94%) patients achieved pCR, 76 (46.06%) patients were evaluated as having a PR, 18 (10.91%) patients were evaluated as SD, and 15 (9.09%) patients experienced PD. To stratify the patients according to either full or anatomically limited (breast-only or node-only) pCR, 56 (33.94%) of patients achieved full (both breast and node) pCR, 10 (6.06%) of patients achieved breast-only pCR, and 39 (23.64%) of patients achieved node-only pCR. The median follow-up time for the entire cohort was 41 months (1–74) months. In total, 48 patients experienced recurrence or metastasis, and 34 of them progressed to death even receiving multiple lines of systemic treatment after relapse. The first relapsed site of these 48 patients and their prognoses are described in **Table 1**. Relapsed patients experienced a really poor outcome, and the mortality rate was extremely high if the relapse site involved the bone or visceral organs.

**Table 1:** First relapsed site and survival outcome.

The first relapsed site	Patients	
	Death (n = 34)	Survival (n = 14)
	n (%)	n (%)
Brain	0 (0.00)	1 (100.00)
Soft Tissue (Chest wall, Breast, Nodes)	8 (50.00)	8 (50.00)
Bone (Vertebral, Pelvis)	5 (83.30)	1 (16.70)
Viscera (Liver, Lung, Pleura)	21 (84.00)	4 (16.00)

**Figure 1:**
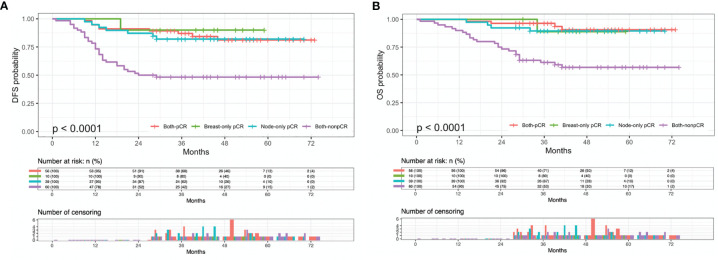
Kaplan-Meier plots of **(A)** disease-free survival (DFS) and **(B)** overall survival (OS) in both-pCR, breast-only pCR, node-only pCR, and both-nonpCR groups. pCR, pathologic complete response.

The mean DFS rates for the pCR, PR, SD, and PD groups were 64.0, 59.5, 39.1, and 27.2 months (p < 0.001), and the mean OS rates were 69.1, 64.6, 49.0, and 35.5 months (p < 0.001), respectively (**Supplementary Figure 1**). Stratifying cases with either full or limited pCR, the mean DFS of both-pCR, breast-only pCR, node-only pCR and both non-pCR groups were 64.0, 55.0, 60.6 and 42.4 months (p < 0.001), the mean OS rates were 69.1, 56.2, 65.0, and 51.1 months (p < 0.001), respectively (**Figure 1**).

### Clinicopathologic Characteristics of Training and Validation Cohorts

For nomogram construction and validation, the entire cohort was randomly (4:1) divided into training (132 patients) and validation (33 patients) cohorts. The clinicopathological characteristics of the training and validation cohorts before NACT and after surgery are summarized in **Table 2**. As for the overall cohort, the mean age was 48.16 ± 10.01 (mean ± standard deviation, the same below) years, 21.21% of patients were no older than 40 years, 57.58% of patients were premenopausal, and 12.73% of patients had a family history of breast cancer. The mean tumor size was 4.58 ± 2.48 cm. Most patients were invasive ductal carcinoma (IDC), while others were lobular, metaplastic and micropapillary carcinoma. Half of the cohort was histological grade 2. Most (81.21%) patients were high expression of Ki-67 (> 29%) and 106 (64.24%) patients were AR negative. Most patients completed full cycles of NACT before surgery while others did not because tumor recession fulfilled the purpose of NACT (breast conserving surgery or downstaging for operable) and they received the remaining planned NACT cycles after surgery. Surgical specimens were thoroughly examined and evaluated. The mean residual tumor size was 1.84 ± 2.73 cm. LVI was identified in 29 patients. Only 8 (4.85%) patients received standard capecitabine treatment for intensive adjuvant chemotherapy. There were no significant differences between the training and validation cohorts.

**Table 2:** Clinicopathologic characteristics for the training and validation cohorts of 165 TNBC patients.

Clinicopathologic characteristics	﻿Overall cohort (n = 165)	﻿Training cohort (n =132)	﻿Validation cohort (n = 33)	p
	n (%)	n (%)	n (%)	
**Age groups (years)**				
≤40	35 (21.21)	29 (21.97)	6 (18.18)	0.634
>40	130 (78.79)	103 (78.03)	27 (81.82)	
**Menopausal status**				
Premenopausal	95 (57.58)	73 (55.30)	22 (66.67)	0.237
Postmenopausal	70 (42.42)	59 (44.70)	11 (33.33)	
**Family history**				
No	144 (87.27)	118 (89.39)	26 (78.79)	0.140
Yes	21 (12.73)	14 (10.61)	7 (21.21)	
**Tumor size (cm)**	4.58 ± 2.48	4.63 ± 2.53	4.40 ± 2.32	0.635
**﻿Clinical tumor stage**				
cT1	16 (9.70)	13 (9.85)	3 (9.09)	0.666
cT2	95 (57.58)	78 (59.09)	17 (51.52)	
cT3	38 (23.03)	30 (22.73)	8 (24.24)	
cT4	16 (9.70)	11 (8.33)	5 (15.15)	
﻿**Clinical nodal stage**				
cN0	23 (13.94)	20 (15.15)	3 (9.09)	0.664
cN1	77 (46.67)	59 (44.70)	18 (54.55)	
cN2	42 (25.45)	35 (26.52)	7 (21.21)	
cN3	23 (13.94)	18 (13.64)	5 (15.15)	
**Histology type**				
IDC	148 (89.70)	119 (90.15)	29 (87.88)	0.750
Others	17 (10.30)	13 (9.85)	4 (12.12)	
**Histological grade**				
Grade 2	94 (56.97)	77 (58.33)	17 (51.52)	0.479
Grade 3	71 (43.03)	55 (41.67)	16 (48.48)	
**Ki-67**				
≤ 29%	31 (18.79)	21 (15.91)	10 (30.30)	0.079
> 29%	134 (81.21)	111 (84.09)	23 (69.70)	
**AR**				
Negative	106 (64.24)	86 (65.15)	20 (60.61)	0.626
Positive	59 (35.76)	46 (34.85)	13 (39.39)	
**NACT cycles**				
Incomplete	36 (21.82)	28 (21.21)	8 (24.24)	0.706
Completed	129 (78.18)	104 (78.79)	25 (75.76)	
**Residual tumor size (cm)**	1.84 ± 2.73	1.64 ± 2.39	2.65 ± 3.74	0.058
**ypT stage**				
ypT0/is	66 (40.00)	55 (41.67)	11 (33.33)	0.591
ypT1	50 (30.30)	41 (31.06)	9 (27.27)	
ypT2	37 (22.42)	27 (20.45)	10 (30.30)	
ypT3	12 (7.27)	9 (6.82)	3 (9.09)	
**ypN stage**				
ypN0	95 (57.58)	80 (60.61)	15 (45.45)	0.420
ypN1	23 (13.94)	18 (13.64)	5 (15.15)	
ypN2	22 (13.33)	16 (12.12)	6 (18.18)	
ypN3	25 (15.15)	18 (13.64)	7 (21.21)	
**LVI**				
Negative	125 (75.76)	103 (78.03)	22 (66.67)	0.173
Positive	40 (24.24)	29 (21.97)	11 (33.33)	
**Standard capecitabine treatment**				
No	157 (95.15)	125 (94.70)	32 (96.97)	1.000
Yes	8 (4.85)	7 (5.30)	1 (3.03)	

TNBC, triple negative breast cancer; IDC, invasive ductal carcinoma; ﻿AR, androgen receptor; NACT, neoadjuvant chemotherapy; LVI, lymphatic vessel invasion; RCB, residual cancer burden; pCR pathologic complete response; PR, partial response; SD, stable disease; PD, progressive disease.

### Variable Selection and Nomogram Establishment

**Figure 2:**
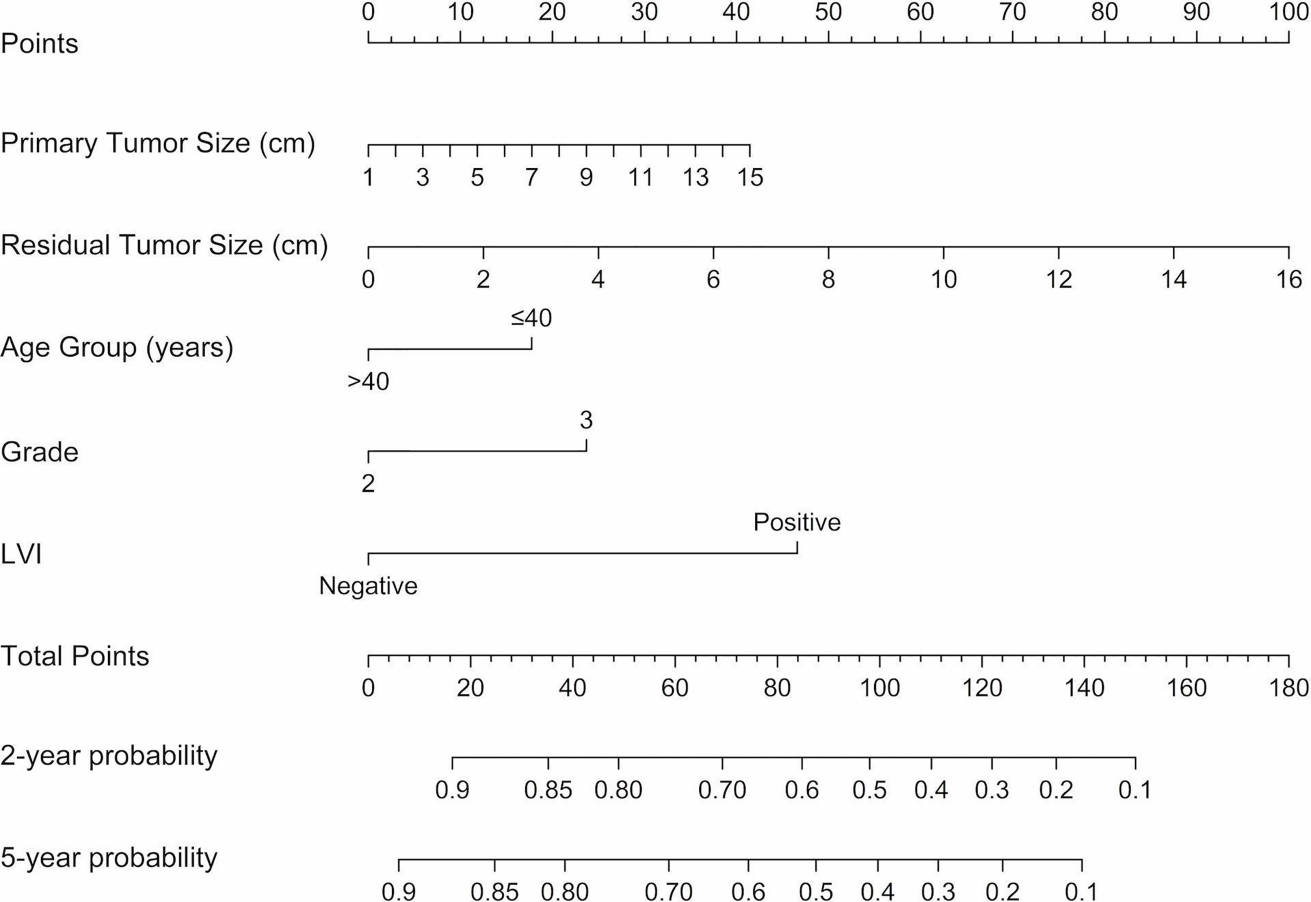
Nomograms predicting 2-year and 5-year DFS following neoadjuvant chemotherapy for TNBC. A vertical line is drawn from each variable to the points scale, then all the five points are summed and a vertical line is drawn from the total points scale to the 2-year and 5-year DFS scale to obtain the likelihood of 2-year or 5-year disease-free survival. LVI, lymphatic vessel invasion; DFS, disease-free survival.

Cox regression analysis was performed for the univariate and multivariate analyses in the training cohort (**Table 3**). Univariate Cox regression analyses revealed younger age (≤40), larger primary tumor size, late clinical tumor stage, other histological types, higher histological grade, larger residual tumor size, late ypT stage, late ypN stage, and the presence of LVI was associated with a higher rate of disease relapse. Among these variables, clinical tumor stage and ypT were removed for multivariate analyses because linearly correlated with primary tumor size and residual tumor size. Other variables were included in the multivariate Cox regression analyses based on lower AIC and BIC criteria. Five variables. including age groups, primary tumor size, histological grade, residual tumor size, and LVI were left for the construction of the nomogram (C-index = 0.815, 95% CI: 0.779-0.851, AIC = 307.379, BIC = 315.156). In addition, a larger residual tumor size (HR 1.175, 95% CI 1.038-1.331, p = 0.011) and the presence of LVI (HR 3.168, 95% CI 1.558-6.441, p = 0.001) were identified as independent predictors of disease relapse. The nomogram was constructed to predict 2-year and 5-year DFS based on the prognostic factors identified in the training cohort (**Figure 2**).

**Table 3:** Cox proportional hazards regression analyses of disease relapse in the training cohort (132 cases).

Variable	Events	﻿Univariate analysis	p	Multivariate analysis	p
	n (%)	HR (95% CI)		HR (95% CI)	
**Age (years)**	35 (26.52)	0.983 (0.952, 1.015)	0.300		
**Age groups (years)**					
≤40	12 (41.38)	1.000		1.000	
>40	23 (22.33)	0.475 (0.236, 0.956)	0.037^*^	0.664 (0.319, 1.383)	0.274
**Menopausal status**					
Premenopausal	19 (26.03)	1.000			
Postmenopausal	16 (27.12)	1.074 (0.552, 2.088)	0.834		
**Family history**					
No	33 (27.97)	1.000			
Yes	2 (14.29)	0.487 (0.117, 2.030)	0.323		
**Tumor size (cm)**	35 (26.52)	1.174 (1.058, 1.302)	0.002^**^	1.071 (0.939, 1.221)	0.309
**﻿Clinical tumor stage**					
cT1	1 (7.69)	1.000			
cT2	19 (24.36)	3.437 (0.460, 25.682)	0.229		
cT3	9 (30.00)	4.575 (0.579, 36.125)	0.149		
cT4	6 (54.55)	10.015 (1.204, 83.331)	0.033^*^		
﻿**Clinical nodal stage**					
cN0	4 (20.00)	1			
cN1	17 (28.81)	1.439 (0.484, 4.279)	0.512		
cN2	8 (22.86)	1.155 (0.348, 3.838)	0.814		
cN3	6 (33.33)	1.799 (0.508, 6.377)	0.363		
**Histology type**					
IDC	29 (24.37)	1.000			
Others	6 (46.15)	2.410 (0.999, 5.812)	0.049^*^		
**Histological grade**					
Grade 2	14 (18.18)	1.000		1.000	
Grade 3	21 (38.18)	2.487 (1.264, 4.895)	0.008^**^	1.728 (0.834, 3.580)	0.141
**Ki-67**					
≤ 29%	8 (38.10)	1.000			
> 29%	27 (24.32)	0.551 (0.250, 1.216)	0.140		
**AR**					
Negative	23 (26.74)	1.000			
Positive	12 (26.09)	1.001 (0.498,2.013)	0.997		
**NACT cycles**					
Incomplete	9 (32.14)	1.000			
Completed	26 (25.00)	0.725 (0.340, 1.550)	0.408		
**Residual tumor size (cm)**	35 (26.52)	1.283 (1.160, 1.419)	<0.001^***^	1.155 (1.017, 1.312)	0.027*
**ypT stage**					
ypT0/is	8 (14.55)	1.000			
ypT1	11 (26.83)	1.994 (0.801, 4.961)	0.138		
ypT2	11 (40.74)	3.401 (1.367, 8.463)	0.009^**^		
ypT3	5 (55.56)	6.630 (2.163, 20.320)	<0.001^***^		
**ypN stage**					
ypN0	13 (16.25)	1.000			
ypN1	4 (22.22)	1.420 (0.463, 4.356)	0.540		
ypN2	9 (56.25)	4.820 (2.053, 11.317)	< 0.001^***^		
ypN3	9 (50.00)	4.112 (1.752, 9.648)	0.001^**^		
**LVI**					
Negative	18 (17.48)	1.000		1.000	
Positive	17 (58.62)	4.446 (2.282, 8.663)	<0.001^***^	2.931 (1.419, 6.058)	0.004^**^
**Standard capecitabine treatment**					
No	33 (26.40)	1.000			
Yes	2 (28.57)	1.136 (0.272, 4.735)	0.862		

^*^p < 0.05, ^**^p < 0.01, ^***^p < 0.001

﻿HR, hazard ratio; CI, confidence interval; IDC, invasive ductal carcinoma; ﻿AR, androgen receptor; NACT, neoadjuvant chemotherapy; LVI, lymphatic vessel invasion.

### Nomogram Validation

**Figure 3:**
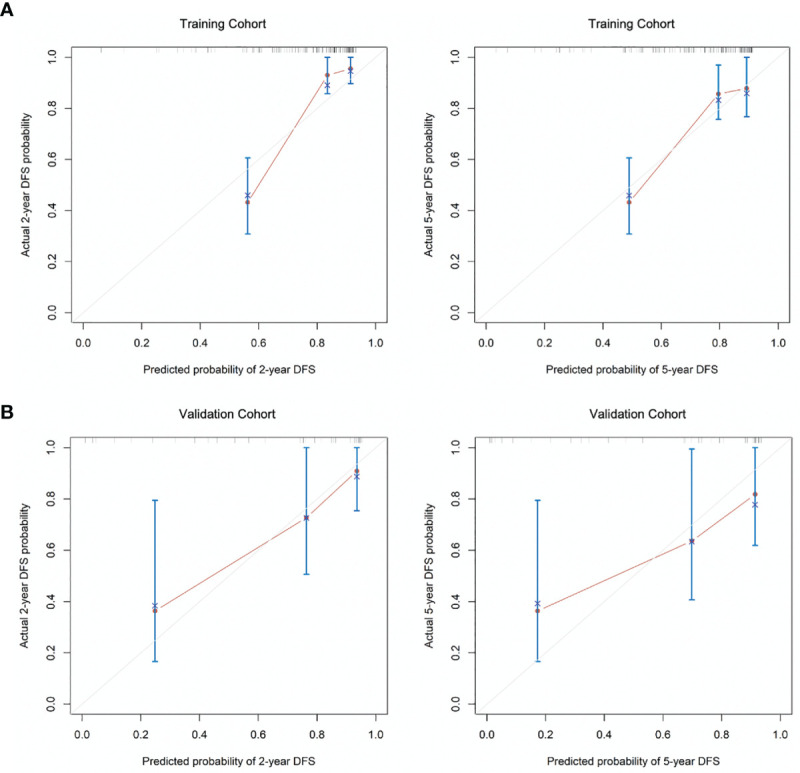
The calibration curves for the **(A)** 2-year and 5-year DFS as the internal validation group derived from the training cohort and **(B)** 2-year and 5-year DFS as external calibration derived from the validation cohort. DFS, disease-free survival.

The C-index for DFS prediction in the training group was 0.815 (95% CI: 0.779-0.851), showing quite acceptable discriminative ability of the nomogram. Then the internal and external calibration curves were created based on the training and validation cohort, respectively, which demonstrated an acceptable consistency between the actual and nomogram-predicted 2-year and 5-year DFS (**Figure 3**).

### ROC Analysis of the Constructed Nomogram Compared With the RCB and 8th AJCC ypTNM Tumor Grade

**Figure 4:**
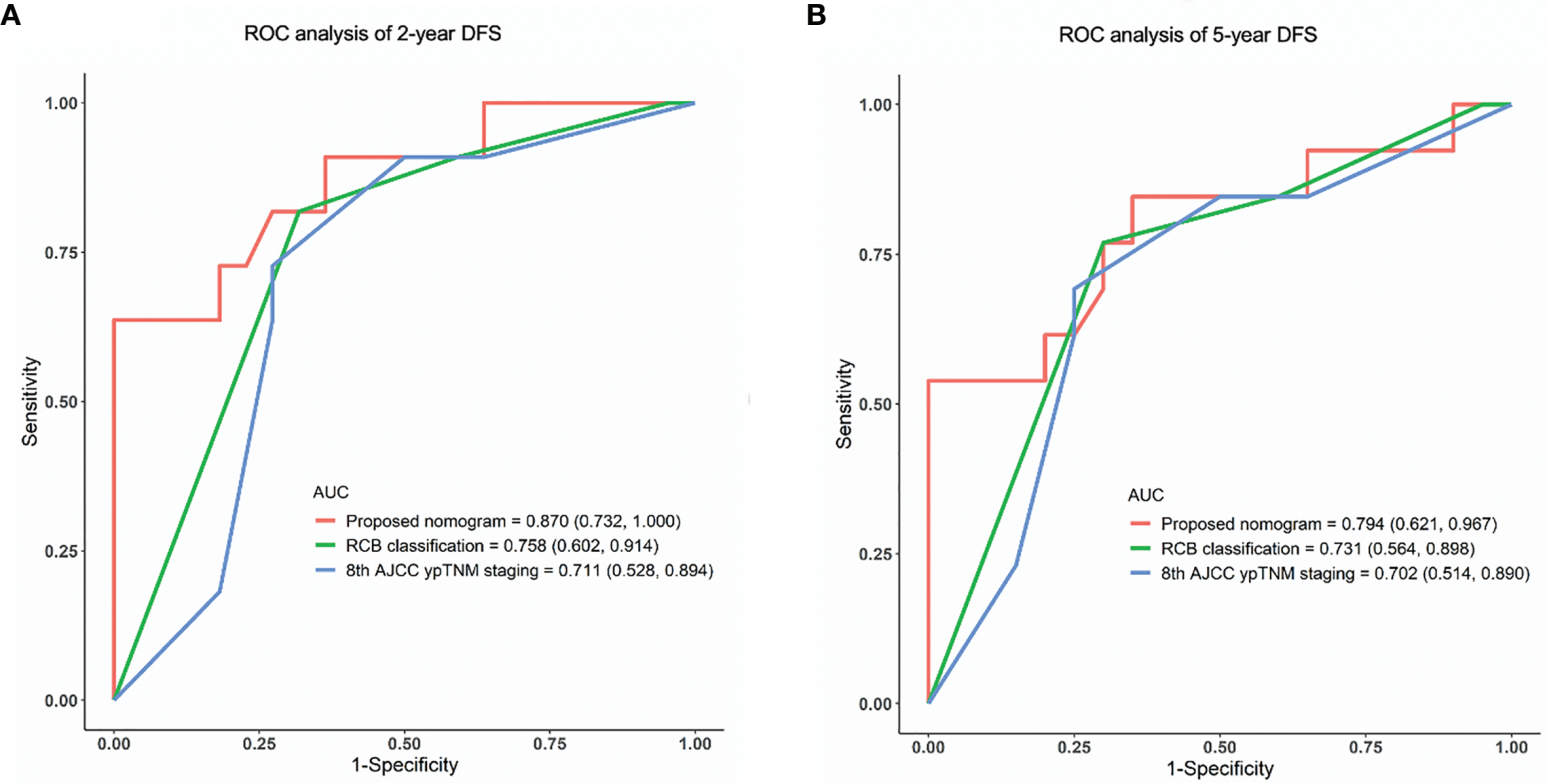
ROC analysis of **(A)** 2-year and **(B)** 5-year DFS in the validation cohort using the proposed nomogram, RCB and 8th AJCC ypTNM system. ROC, receiver operating characteristic; AUC, area under the curve; DFS, disease-free survival.

The accuracy and probability of our nomogram was compared with the RCB and 8th AJCC ypTNM classification system using ROC analysis. The time-dependent ROC curves for predicting the 2-year and 5-year DFS in the validation cohort are presented in **Figure 4**. ROC curves for the 2-year DFS revealed that the present nomogram had a higher AUC of 0.870 (95% CI: 0.732-1.000), which was superior to that of the RCB and 8th AJCC ypTNM system with an AUC of 0.758 (95% CI: 0.602-0.914) and 0.711 (95% CI: 0.528-0.894), respectively (**Figure 4A**). Similarly, the ROC curves for the 5-year DFS showed that the constructed nomogram had a higher AUC of 0.794 (95% CI: 0.621-0.967), which was superior to that of the RCB and 8th AJCC ypTNM classification systems having an AUC of 0.731 (95% CI: 0.564-0.898) and 0.702 (95% CI: 0.514-0.890), respectively (**Figure 4B**). These results indicated that the present nomogram had an improved predictive ability and discrimination.

## Discussion

The present study established and validated a practical nomogram to predict 2-year and 5-year DFS based on clinicopathological characteristics for TNBC patients who underwent a standard regimen of NACT accompanied by a combination of anthracyclines and taxanes. The constructed nomogram integrates five variables, including age groups, primary tumor size, histological grade, residual tumor size, and LVI. Among these variables, residual tumor size and LVI were independent risk factors for disease recurrence. The nomogram demonstrated superior prognostication performance compared with the RCB classification and the 8th AJCC ypTNM staging system (AUC of 2-year DFS ROC curve, 0.870 *vs.* 0.758 *vs.* 0.711, respectively; AUC of 5-year DFS ROC curve, 0.794 *vs.* 0.731 *vs.* 0.702, respectively).

The pCR is a strong surrogate for aggressive phenotypes of breast cancer, such as TNBC and HER-2 positive breast cancer, and the prognosis of patients who obtained pCR after NACT can be significantly improved (3). In this study, the DFS and OS of patients who achieved pCR were significantly improved compared with other groups. After stratifying patients according to either full or anatomically limited (breast-only or node-only or both disease) pCR, patients who achieved either full or anatomically limited pCR also had a significantly improved DFS and OS than patients who did not. Notably, for TNBC patients, who have fewer treatment options after NACT than the other phenotypes, these partial and complete responders represent a special group, with a favorable response to systemic therapy and the ability to overcome unfavorable tumor biology (15). However, the prognosis of TNBC is reported to be extremely poor once recurrence and metastasis have occurred (16). In our study, after a follow-up of a median of 41 months, 48 patients of the cohort experienced recurrence or metastasis, and bone or visceral metastasis were associated with extremely high mortality. Notably, one of these patients was identified as solitary brain metastasis. She underwent neurosurgery for metastatic foci resection combined with systemic therapy and is still alive at the time of writing without disease progression for more than 25 months since surgery. Therefore, it can be inferred that for oligometastatic brain of TNBC, if evaluated as Recursive Partitioning Analysis Radiation Therapy Oncology Group (RPA RTOG) prognostic class I, intensive local and systemic treatment had a strong survival benefit (17). However, more studies are required for further confirmation of strategy for these oligometastatic brain patients.

Age may be a prognostic factor for breast cancer. According to the 3rd International Consensus Conference for Breast Cancer in Young Women (BCY3), a greater proportion of TNBC and HER-2 positive disease and more advanced stages, with higher recurrence rate, and worse prognosis is observed in young breast cancer patients (≤40 years) than older patients (18). A previous study in China has reported that the incidence of young breast cancer (≤39 years) accounts for about 10% in TNBC, accompanied with frequent pathogenic germline variants and predominant homologous recombination deficiency, resulting worse short-term survival time (19). In this study, young breast cancer (≤40 years) accounted for 21.21% in TNBC. Younger age showed a significant correlation with predicting disease relapse, but it was not an independent prognostic factor. However, it was included in the nomogram for DFS prediction as younger age may be a high-risk factor for TNBC. Regretfully, data on germline mutation status was unavailable for the majority of this cohort. Further studies are needed to explore the role of age and germline mutation status in survival prognosis.

Ki-67 is considered to be an important proliferation marker of tumor cells (20). The proposed cut-off values for Ki-67 expression to distinguish luminal A and luminal B phenotypes has gone through several changes. Currently, a cut-off value within the range of 20%-29%, is used as the reference value in our local laboratory as well and has been considered reasonable since the St Gallen Consensus in 2015 (21–23). Many studies used different cut-off values to define high expression of Ki-67 and the majority have indicated that high Ki-67 expression is a predictor of the response to NACT treatment (24). The cut-off value adopted in this study was 29% and the stratification of the patient cohort based on this Ki-67 level was the same as the cohort in previously reported study (25), which revealed that low Ki-67 expression was an independent predictor of PD. However, low Ki-67 levels showed no significant correlation with prognosis.

The AR is a steroid hormonal receptor and acts as a transcription factor that can stimulate or suppress both cell proliferation and apoptosis (26). AR is expressed in about 70%-90% of breast cancers and the immunohistochemical expression level of AR varies from 10% to 90% in TNBC (27). Various studies have reported that the pCR rate was relatively higher in the AR negative group (28, 29). In contrast, AR positivity was associated with reduced chemotherapy responsiveness and lower pCR rate, but better prognosis after neoadjuvant treatment (26). Consistent with these studies, the percentage of AR negative patients in our study was 64.24%. A previous report revealed that AR negative status was an independent predictor of pCR (25). Nevertheless, AR status had no significant correlation with prognosis in Cox regression.

LVI refers to the presence of tumor emboli in lymphatic spaces, blood vessels, or both within the peritumoral area. The prognostic value of LVI in breast cancer has been extensively studied and the majority of studies have considered LVI as a marker of increased risk of axillary nodal metastases and unfavorable survival outcome in all breast phenotypes except in the HER2-positive subgroup (30). Although it has not been incorporated into most international breast cancer staging systems, the presence of LVI should be recorded in the pathological report of surgical specimens following NACT. Even if LVI is the only residual disease in the breast after NACT, the response to chemotherapy cannot be diagnosed as pCR (31), which provides evidence supporting the influence of LVI on the survival prognosis after NACT for breast cancer. In this study, patients with LVI-positive tumors following NACT represented 24.24% of the entire cohort. Additionally, consistent with previous studies, LVI positivity was an independent predictor of relapse after NACT. Thus, the present nomogram including LVI is more comprehensive and may supplement the RCB classification and AJCC ypTNM staging system.

The CREATE-X study showed that intensive capecitabine therapy should be recommended in the adjuvant stage for HER-2 negative patients with residual invasive disease after standard NACT to improve prognosis, especially in patients with TNBC (4). Based on these findings, the 3rd edition of the National Comprehensive Cancer Network (NCCN) guideline in 2017 was updated to include capecitabine adjuvant therapy in TNBC and residual disease after standard NACT, which was re-classified as category 2A (32). Two thirds of the cohort in this study was diagnosed before 2017, thus only 33 (30.28%) patients with non-pCR in this study received capecitabine following NACT. Additionally, because of concerns regarding the hand-foot syndrome and hematological toxicity due to capecitabine, the majority of patients were reluctant to insist on treatment with capecitabine for 6-8 courses. Hence, only 8 patients received standard course treatment of capecitabine in this study, and as a result, there was no significant correlation between standard capecitabine treatment and DFS. Nevertheless, our institution still recommends capecitabine as intensive adjuvant therapy for non-pCR TNBC patients, with reference to the NCCN guideline.

Although the clinicopathological factors mentioned herein have been studied in previous prediction models of pCR, the present study is the first to combine them together to establish a 2-year and 5-year DFS nomogram. All of these predictors are easy to obtain, hence, the proposed nomogram can be used as a practical tool for clinicians to guide routine follow-up of TNBC patients after NACT. For patients with a high recurrence score predicted by the nomogram, closer follow-up and necessary extra imaging studies could be recommended. In addition, intensive adjuvant therapy of capecitabine could be proposed. Despite the nomogram showing better predictive performance than the existing prognostic system, there are still some limitations. First, as the data in the nomogram was retrospectively collected, some important factors such as genetic data, histological grade information before NACT, were not available. Most patients lack information on germline mutation status; thus, this important factor was not incorporated into the nomogram, which may lead to a predictive bias. Second, once disease relapse occurred, patients in this study received multiple lines of treatment without standard regimens, some received traditional chemotherapy and others participated in clinical trials. Therefore, it is difficult to construct a nomogram for predicting OS. Third, limited by its single-center retrospective data design, the predictive efficacy of the nomogram needs to be confirmed and optimized in a multi-center prospective cohort.

## Conclusion

This study constructed and validated a nomogram to predict 2-year and 5-year DFS for TNBC patients following standard NACT based on easy-to-obtain clinicopathological factors, which included age, primary tumor size, histological grade, residual tumor size, and LVI status. The nomogram demonstrated a relatively higher predictive ability than the RCB classification or the 8th AJCC ypTNM staging system. Our nomogram can be used as an easy-to-use tool for clinicians to guide individualized surveillance of TNBC patients following standard NACT.

## Data Availability Statement

The raw data supporting the conclusions of this article will be made available by the authors, without undue reservation.

## Ethics Statement

The studies involving human participants were reviewed and approved by Clinical Research Ethics Committee of Zhejiang Cancer Hospital(IRB-2020-327). The patients/participants provided their written informed consent to participate in this study.

## Author Contributions

MZ and DC conceptualized and designed the work. MZ collected, analyzed, and interpreted the data, and drafted the article. LZ checked pathology data. CL and FZ collected and analyzed the data. DC critically revised the article and gave the final approval of the version to be published. All authors contributed to the article and approved the submitted version.

## Funding

This work was supported by grants from the Medical Health Science and Technology Project of Zhejiang Provincial Health Commission (2021RC045). The funder had no role in the study design, data collection and analysis, decision to publish, or preparation of the manuscript.

## Conflict of Interest

The authors declare that the research was conducted in the absence of any commercial or financial relationships that could be construed as a potential conflict of interest.

## Publisher’s Note

All claims expressed in this article are solely those of the authors and do not necessarily represent those of their affiliated organizations, or those of the publisher, the editors and the reviewers. Any product that may be evaluated in this article, or claim that may be made by its manufacturer, is not guaranteed or endorsed by the publisher.

## Supplementary Material

Supplementary Figure 1 Kaplan-Meier plots of **(A)** disease-free survival (DFS) and **(B)** overall survival (OS) rates in the pCR, PR, SD, and PD groups. pCR, pathologic complete response; PR, partial response; SD, stable disease; PD, progressive disease.
