# Supplementary figures and images for: A Nomogram to Predict Disease-Free Survival Following Neoadjuvant Chemotherapy for Triple Negative Breast Cancer
Source: Front Oncol. 2021 Oct 21;11:690336. doi: 10.3389/fonc.2021.690336 (PMC8566908; doi:10.3389/fonc.2021.690336)

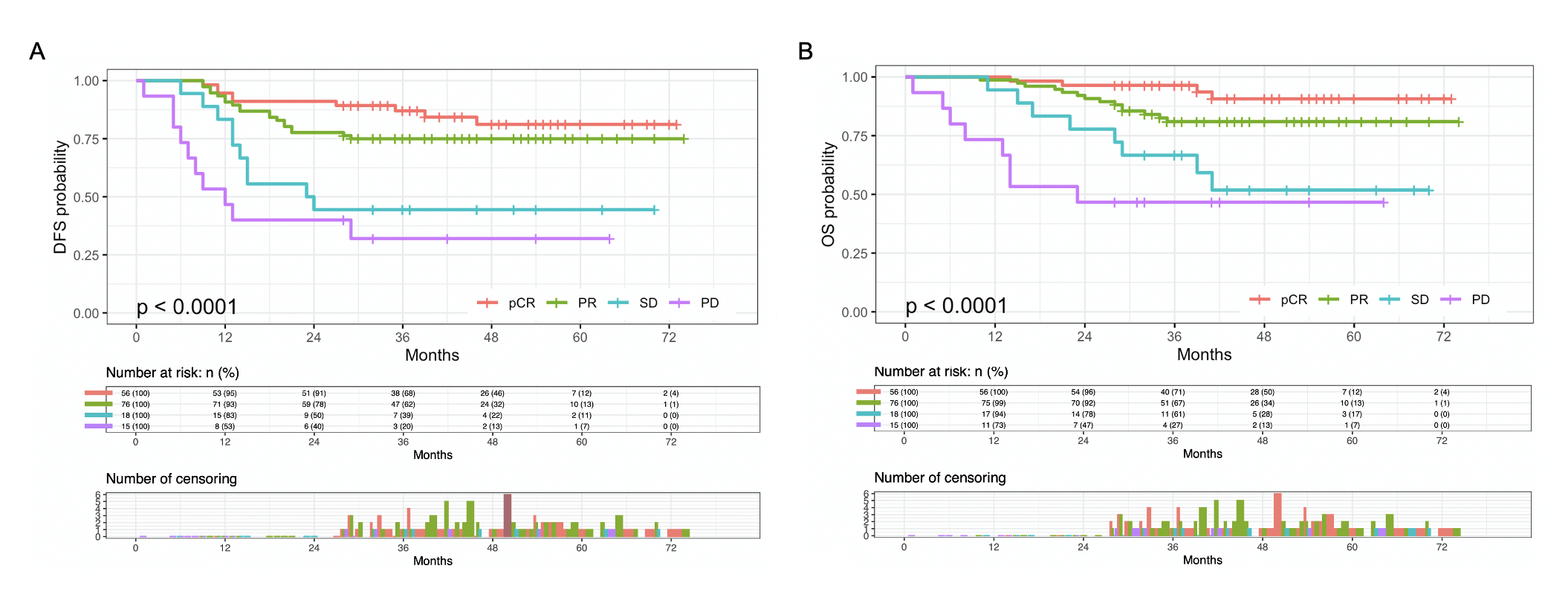

Supplement: Supplementary Figure 1 — Kaplan-Meier plots of (A) disease-free survival (DFS) and (B) overall survival (OS) rates in the pCR, PR, SD, and PD groups. pCR, pathologic complete response; PR, partial response; SD, stable disease; PD, progressive disease. [file Image_1.tiff]
